# Biomechanical and physiological effects of passive upper limb exoskeletons in simulated manufacturing tasks

**DOI:** 10.1017/wtc.2025.10021

**Published:** 2025-08-01

**Authors:** Francesco Scotto di Luzio, Christian Tamantini, Raffaele Di Maro, Chiara Carnazzo, Stefania Spada, Francesco Draicchio, Loredana Zollo

**Affiliations:** 1 Research Unit of Advanced Robotics and Human-Centred Technologies, Università Campus Bio-Medico di Roma, Rome, Italy; 2 Institute of Cognitive Sciences and Technologies, National Research Council of Italy, Rome, Italy; 3 Stellantis SpA, Turin, Italy; 4 Department of Occupational and Environmental Medicine, INAIL, Rome, Italy

**Keywords:** biomechanical workload, musculoskeletal disorders, physiological workload, human-robot collaboration

## Abstract

In the last two decades, the adoption of exoskeletal devices for the reduction of the biomechanical overload of workers has hugely increased. They allow relief of the biomechanical load of the operator and ensure the operator’s contact with the object without binding its interaction. In this work, the biomechanical and physiological effects on the user wearing upper limb passive exoskeletons have been evaluated to highlight the benefits and possible drawbacks introduced by their use in typical manufacturing tasks. MATE and PAEXO Shoulder passive exoskeletons have been assessed during the execution of different working gestures among static, dynamic, and quasi-static tasks on 16 healthy volunteers. The obtained results confirm that the adoption of such systems significantly impacts the users by reducing the muscular load, increasing endurance, and reducing the perceived effort. Moreover, this analysis pointed out the specific benefits introduced by one exoskeleton with respect to the other according to the specific task. The MATE has the potential to reduce muscle load during the execution of static tasks. Conversely, the PAEXO Shoulder positively impacts the users’ biomechanical performances in dynamic tasks.

## 1. Introduction

The advent of the new technologies in the Industry 4.0 allowed a clear improvement in working conditions. Up to 10 years ago, the assembly of a new product and all its parts was carried out by workers, often with considerable physical effort and non-ergonomic postures. Nowadays, many parts of this assembly line have been almost fully automated (Bragança et al., 2019). However, in many processes, the human operator is still indispensable and is an integral part of the production linked to the Industry 4.0 landscape (Munoz, 2018). It has been shown in the state of the art that the assumption of incongruous postures, the execution of repetitive movements, and the manipulation of heavy as well as light loads at high frequency can lead to the onset of work-related musculoskeletal diseases, which have a high cost and represent one of the major causes of absence from work. The risk of shoulder injuries is particularly high during activities that involve raising the upper limbs above the shoulder, commonly performed in the construction and manufacturing sectors (McAtamney and Corlett, 1993). Furthermore, it has been demonstrated that even during low-level sustained contractions ( 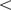 10% of the maximum voluntary contraction [MVC]), usually sufficient to prevent fatigue, MVC decreases to 10% already starting from 1 hour of contraction at 5% MVC (Sjpgaard et al., 1988). For these reasons, the introduction of collaborative robots and exoskeletons could represent an effective solution to stem the onset of such disturbances (De Looze et al., 2016; Dahmen and Constantinescu, 2020).

The adoption of exoskeletal devices for the reduction of biomechanical overload of the worker is already widely attested because they allow relief of the biomechanical load of the operator, due to the particular mechanical structure of the robot, and guarantee the operator’s contact with the object without binding its interaction (Dimitrov et al., 2006). Exoskeletons are typically classified into active and passive. An exoskeleton equipped with powered actuators is defined as active; on the other hand, if it consists of passive mechanical elements, such as spring systems capable of providing support, they are defined as passive. Besides these, semi-passive exoskeletons, which lie between the preceding classifications, adopt low power components and are able to provide a higher level of flexibility yet need active user input and interaction to operate properly (Crea et al., 2021; Grazi et al., 2020; Herr, 2009).

Commercial passive exoskeletons are commonly adopted in the manufacturing context. There are several examples of robotic systems for shoulders and upper limb, such as Shoulder X (SuitX, USA), MATE exoskeleton (COMAU, Italy), Airframe (Levitate Technologies, USA), and Paexo Shoulder (Ottobock, Germany) conceived to support the worker in performing overhead tasks and repetitive movements of the arms with a compact design and able to adapt to the anatomy of the limb, providing gravity compensation that reduces the load on the muscles of the limb. Such exoskeletons adhere to the upper arms and lower back, which, during work activities, transfers the load of the arms from the shoulders to the frame on the back. The support torque varies with the elevation angle of the arm and the torque due to the action of gravity on the arm to obtain transparent assistance.

Evidently, several factors can affect the effectiveness of such systems. An exoskeleton can limit and/or modify the kinematics of movements, altering the biomechanics, influencing postural effort, and compromising the user’s physiological response. Exoskeletons can alter the range of motion and posture during tasks. While some studies found minimal changes in joint angles and posture (Iranzo et al., 2020), others reported significant differences in upper limb kinematics between exoskeleton and non-exoskeleton conditions (Theurel et al., 2018). These changes are generally small but highlight the need for careful design to ensure natural movement patterns are maintained. Passive exoskeletons significantly reduce muscle activity in the shoulder area, particularly in the anterior and medial deltoid muscles, during industrial tasks. Studies report reductions ranging from 21.6% to 34%, contributing to decreased discomfort and fatigue during prolonged activities (McFarland and Fischer, 2019; Iranzo et al., 2020; Kim et al., 2022). By alleviating the muscular demands on the upper limbs, passive exoskeletons help mitigate the risk of work-related musculoskeletal disorders, especially in tasks involving overhead work (McFarland and Fischer, 2019; Grazi et al., 2020). While passive exoskeletons reduce muscle activity, they can also lead to increased trunk and shoulder flexion angles, which may require users to adjust their posture during tasks like patient handling (Hwang et al., 2021; Erezuma et al., 2023). Furthermore, as shown in (Kim et al., 2018), the use of the ExoVest exoskeleton reduces both maximum and average muscle activity in shoulder-associated muscle groups by approximately 45% and 50%, respectively, across different working heights. However, while exoskeleton use leads to a slight increase in task error, it also shortens overall task duration by 20%. By reducing muscle activity, exoskeletons can potentially enhance endurance and reduce fatigue during prolonged tasks (McFarland and Fischer, 2019).

Furthermore, a very limited number of works evaluated the physiological state of workers during the use of these devices (Weston et al., 2022; Tamantini et al., 2023). Monitoring of physiological parameters can be introduced in the validation of exoskeletal devices as they are able to provide indications of the workload experienced by the operator from both physical and cognitive points of view (Aryal et al., 2017; Lanata et al., 2020). Intense physical activity causes an increase in both the heart and respiration rates (Wulfert et al., 2005; Meng et al., 2014; Theurel et al., 2018). Some studies have reported reductions in heart rate and metabolic parameters, such as oxygen consumption, when using exoskeletons, indicating a decrease in overall physical strain (Grazi et al., 2020; Blanco et al., 2022). Several studies have investigated the impact of passive upper limb exoskeletons on heart rate. Generally, these devices do not significantly alter heart rate during tasks. For instance, one study found no significant difference in heart rate when participants used a passive arm-support exoskeleton during field tasks (Pentenga et al., 2024). Another study reported a reduction in heart rate when using a passive exoskeleton during a carrying task, suggesting a decrease in physical workload (Garcia et al., 2023). Similarly, the H-PULSE semi-passive exoskeleton was shown to reduce heart rate during prolonged static overhead tasks, indicating a potential reduction in cardiovascular strain (Grazi et al., 2020). Another measure that can be used in automation construction is the galvanic skin response since it is capable of providing information about the mental workload required to accomplish a certain task (Tao et al., 2019). The current state of the art does not directly address galvanic skin response (GSR) in the context of passive upper limb exoskeletons. GSR is typically used to measure physiological arousal and stress. Classical conditioning studies have shown that GSR can be influenced by stressors, but this is not directly linked to exoskeleton use. However, in the state of the art, there are only preliminary studies aiming at the evaluation of the effect of passive exoskeletons on the user (Pesenti et al., 2021) and there are no investigations conducted in a systematic way. The effectiveness of the exoskeletal systems and the motor patterns induced by them are typically evaluated through movement analysis techniques, the measurement of the forces involved, and muscle activation (Crea et al., 2021; Pesenti et al., 2021). Ensuring proper alignment between the exoskeleton joints and the worker’s joints is crucial (Pacifico et al., 2020, 2022, 2023; Grazi et al., 2022), as misalignment can generate undesired torques and forces, potentially reducing the expected benefits (Di Natali et al., 2021). The effectiveness of passive exoskeletons varies with the task and posture. For instance, they provide more support during elevated arm tasks and require adjustments in body posture during lifting tasks (de Vries et al., 2019; Luger et al., 2021).

Despite the increasing number of studies on these devices and their potential benefits, there is still a need to assess the effects experienced by users when using different passive exoskeletons under standardized experimental conditions. Evaluating the biomechanical and physiological effects of wearing these devices can reveal both similarities and differences among various robotic systems and tasks, enabling the identification of optimal usage scenarios for each device.

This could represent an important turning point for passive exoskeletons, trying to highlight both the quantitative benefits estimated through additional sensors, such as EMG and M-IMU, and also the subjective perception assessed by means of self-reported questionnaires. Hence, this study aims to assess the biomechanical and physiological impact associated with the use of different passive upper limb exoskeletons in typical industrial tasks. By combining quantitative and qualitative metrics, this study seeks to quantify the benefits and potential drawbacks of exoskeleton-assisted work. Specifically, the study evaluates two passive exoskeletons (that is, Paexo Shoulder and MATE), under standardized laboratory conditions that simulate real-world assembly line tasks. This approach allows for a direct comparison between exoskeleton designs, assessing their impact on muscle activation, movement kinematics, physiological responses, and perceived comfort and usability to generate quantifiable and comparable insights into their effectiveness across different task types. To achieve this, a multimodal assessment was conducted, integrating objective measurements and subjective user feedback. The biomechanical impact was analyzed through electromyography (EMG) to assess muscle activation, inertial measurement units (M-IMUs) to evaluate movement kinematics. Physiological impact was examined using heart rate (HR), heart rate variability (HRV), respiration ratio (RR), and galvanic skin response (GSR). Furthermore, qualitative insights were gathered through questionnaires, providing a comprehensive understanding of perceived comfort, usability, and task feasibility when using different exoskeletons. By systematically comparing task execution with and without the exoskeletons, this study aims to generate evidence-based insights into their effectiveness, usability, and potential ergonomic implications in industrial settings. This study makes a novel contribution by enabling a direct comparison between exoskeleton designs, specifically addressing the diverse requirements of tasks commonly encountered in manufacturing environments. By combining quantitative data with qualitative insights, the study aims not only to deepen the understanding of the biomechanical and physiological effects of exoskeleton use but also to provide valuable information on user perception, comfort, and the practical usability of these devices in industrial settings. The paper is organized as follows: In Section 2, the proposed monitoring system to estimate the effect of the upper limb passive exoskeletons, the experimental setup and protocol are presented. Experimental results are illustrated and discussed in Section 3. Finally, conclusions and future work are reported in Section 5.

## 2. Materials and methods

**Figure 1.**
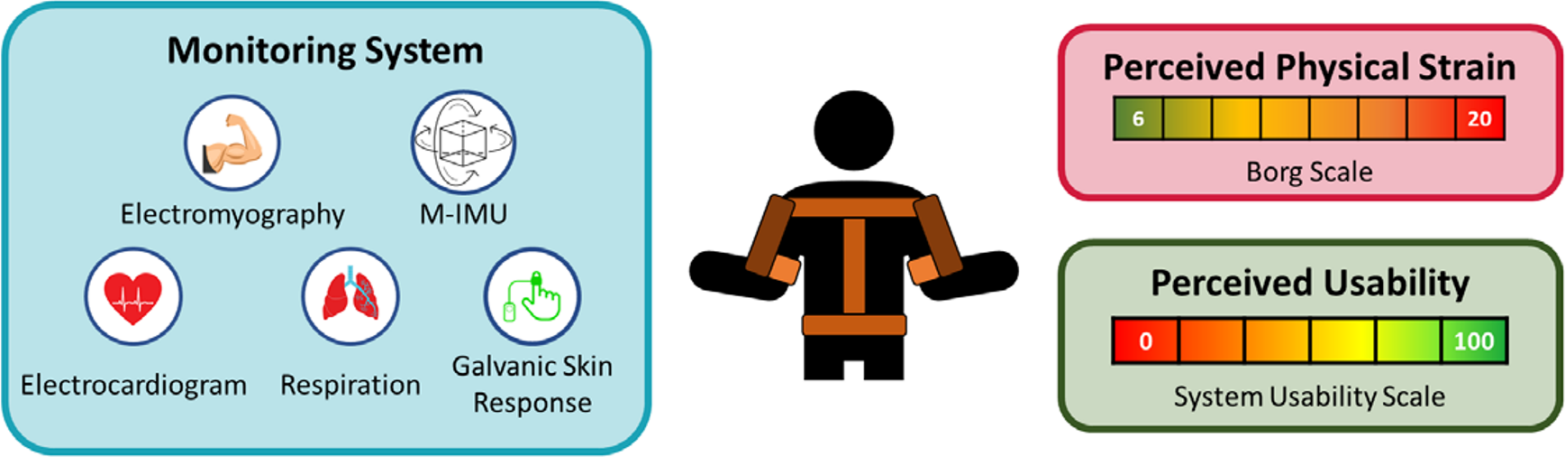
Block scheme of the approach adopted to validate upper limbs passive exoskeletons.

A monitoring system was integrated and tested to objectively measure the effect of the exoskeleton use on the users, reported in Figure 1. Such a platform consists of three data classes: (i) kinematic data (that is, M-IMU) to assess the movements of the user, (ii) physiological data (that is, EMG, cardiac and respiratory activity, and galvanic skin response) to assess the physical and cognitive workload experimented by the enrolled participants, and (iii) self-reported questionnaires to estimate the perceived physical strain and the usability of the robotic system itself. Each block reported in Figure 1 and the exoskeletons are described in depth in the following.

### 2.1. Monitoring system

The monitoring system is conceived for the acquisition of kinematic and physiological data of the user during the execution of static and dynamic tasks with and without the exoskeleton. It is based on yet another robotic platform (Metta et al., 2006) middleware to allow data synchronization, recording, and storage for postprocessing.

**Figure 2.**
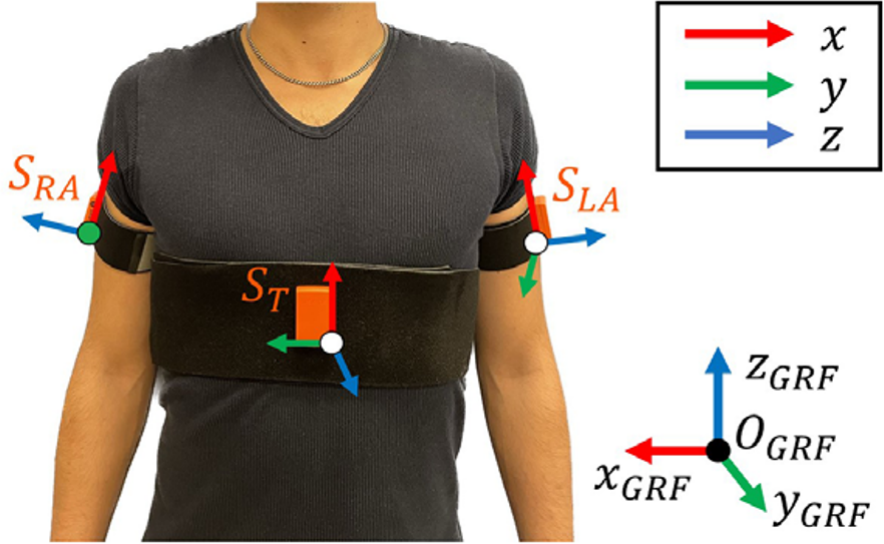
M-IMU positioning on the participants’ body. The global reference frame (GRF) along with the sensor frames are displayed.

More in detail, the shoulder angles were monitored by means of M-IMU sensors. Shoulder kinematic data were retrieved by placing three M-IMU sensors on the users: one sensor has to be positioned on the subject’s trunk 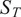 and one sensor per arm ( 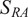 and 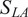 for the right and left arm, respectively), as shown in Figure 2.

Given 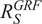 the rotation matrix that expresses the rotation of the sensor 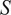 with respect to the Ground Reference Frame ( 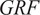 ), the shoulder flexion/extension (sFE) can be obtained as the *Z* angle returned by the inverse problem of Euler angles by following the *ZYX* decomposition (Lapresa et al., 2022). In particular, the rotation matrix of the right shoulder is(1)

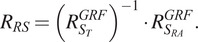



Similarly, the left shoulder rotation can be expressed as(2)

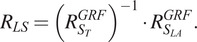



The muscular activity of the user was collected by means of EMG surface electrodes placed according to SENIAM guidelines (Hermens, 1999). Such signals were acquired with a sampling frequency of 1000 Hz, filtered by a band-pass filter with upper and lower cut-off frequencies of 30 and 450 Hz, respectively, and a 50 Hz notch filter. The EMG signals were normalized with respect to the maximum value recorded for each muscle. Additionally, the envelope of the signal was obtained using a 100-ms moving average filter to smooth the data and enhance the accuracy of the analysis.

### 2.2. Upper limb passive exoskeletons

#### 2.2.1. Paexo Shoulder exoskeleton

**Figure 3.**
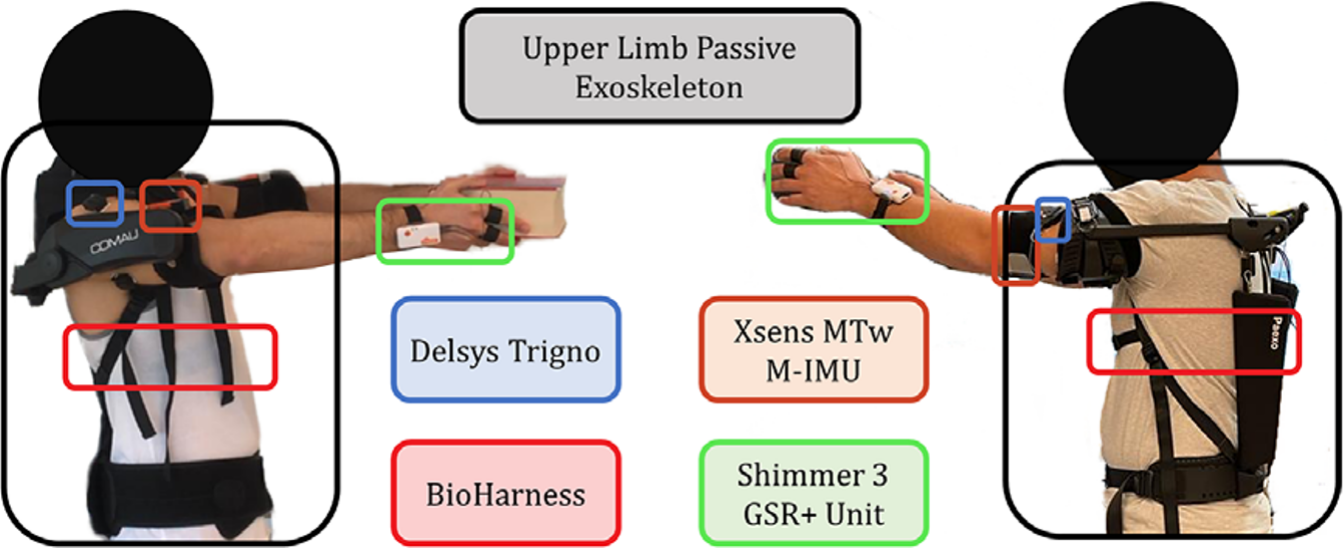
Experimental setup: two representative participants wearing the monitoring system and the passive exoskeleton Paexo and MATE are reported on the right and left side, respectively.

Paexo Shoulder is a commercial passive exoskeleton developed by Ottobock (Germany) for the shoulder and upper limb, as shown in Figure 3. It is designed to generate a supporting torque that varies with the arm elevation angle. It has a lightweight structure (1.9 kg) suitable for the entire work shift. The mechanical structure is designed to be adjustable and follow the movement of the shoulder and its biomechanics. The exoskeleton can be worn like a backpack and adjusted by the belt to allow freedom of movement in all directions of space even though the two ball joints are placed on the hip.

#### 2.2.2. MATE exoskeleton

The MATE system, developed by COMAU (Italy), is a commercial passive exoskeleton for the upper limbs designed to provide support during the execution of work tasks in the manufacturing context, as shown in Figure 3. It has a compact design and is able to adapt to the anatomy of the limb, providing gravity compensation that reduces the load on the muscles of the upper limbs. The MATE exoskeleton features a spring system capable of providing variable assist torque in a manner consistent with the gravity torque profile of the arm in a physiological shoulder lift. The MATE frame consists of a back support structure, which connects the entire device to the user’s body and is made up of shoulder straps, cuffs, and a belt, able to adapt to different anthropometries. The frame is made of aluminum and it is T-shaped, designed to distribute the reaction forces produced by the torque generator in the user’s pelvic area through its belt. The kinematic chain of the exoskeleton allows free movement of the limb and softly counteracts the descent of the limb in flex-extension and ab-adduction movements.

### 2.3. Experimental setup

The proposed monitoring system allowed monitoring kinematic and physiological data. User kinematic was assessed by using XSens MTw M-IMU sensors (Movella, Enschede, The Netherlands). The sampling frequency of the M-IMU sensors was 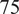 Hz. Delsys Trigno Wireless sensors (Delsys, Massachusetts, USA) were also adopted to monitor the participant’s muscular activity. Fourteen EMG electrodes were placed on the following muscular districts of both the dominant and non-dominant limbs of the user: medial deltoid (MD), anterior deltoid (AD), posterior deltoid (PD), trapezius ascendents (T), triceps brachii (TR), biceps brachii (B), pectoralis major (P).

The monitoring system also includes a physiological sensing unit to collect both vital and non-vital parameters of the users. Cardiorespiratory measurements were carried out by means of the BioHarness 3.0 chest belt, developed by Zephyr™ Technology (Medtronic, Dublin, Ireland). It fuses capacitive and stretch sensors to measure the electrical heart activity and the deformations imposed by the expansions and compressions of the rib cage. To accurately detect these physiological measures, the chest belt has to be worn by the users directly on the skin. The ECG and the breathing waveform are collected with sampling rates of 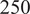 Hz and 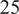 Hz, respectively. Lastly, the galvanic skin response of the users was monitored with the Shimmer 3 GSR+ Unit (Shimmer, Dublin, Ireland), a wearable device whose electrodes are placed on the index and middle fingers of the non-dominant hand. Information about the user’s electrodermal activity is collected with a sampling frequency of 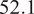 Hz. Figure 3 shows the experimental setup used in the present work. Two representative participants wearing the Paexo Shoulder (the one on the right) and MATE (the one on the left) upper limb passive exoskeletons along with the monitoring system are reported. The placement of the wearable sensors was initially verified to ensure that the exoskeletons would not cause interference during their use. Prior to the experiment, preliminary tests to confirm that the sensor positioning enabled accurate data collection have been conducted, a crucial step to ensure the reliability of the signals and the integrity of the measurements throughout the study. The exoskeletons have been adjusted based on the anthropometry of each volunteer. All supports, belts, pelvic belt and the support level were adjusted to guarantee the maximum comfort for the user, as recommended by their manufacturers and reported in the user manuals. More in detail, the level of assistance provided by the MATE exoskeleton has been tuned according to the height and body mass of the user (Comau, n.d.). The PAEXO has been conceived to generate a support torque that mimics the effect of gravity on the arm. The maximum support torque is provided when the arm is at a 90-degree elevation angle (upper arm horizontal), while the torque is zero when the arm is perpendicular to the ground. As the arm is lowered along the body, the support torque gradually decreases and becomes zero. In addition, PAEXO features an additional adjustment of the passive actuation system to compensate for any external load weight, which was not used in this study.

### 2.4. Experimental protocol

**Figure 4.**
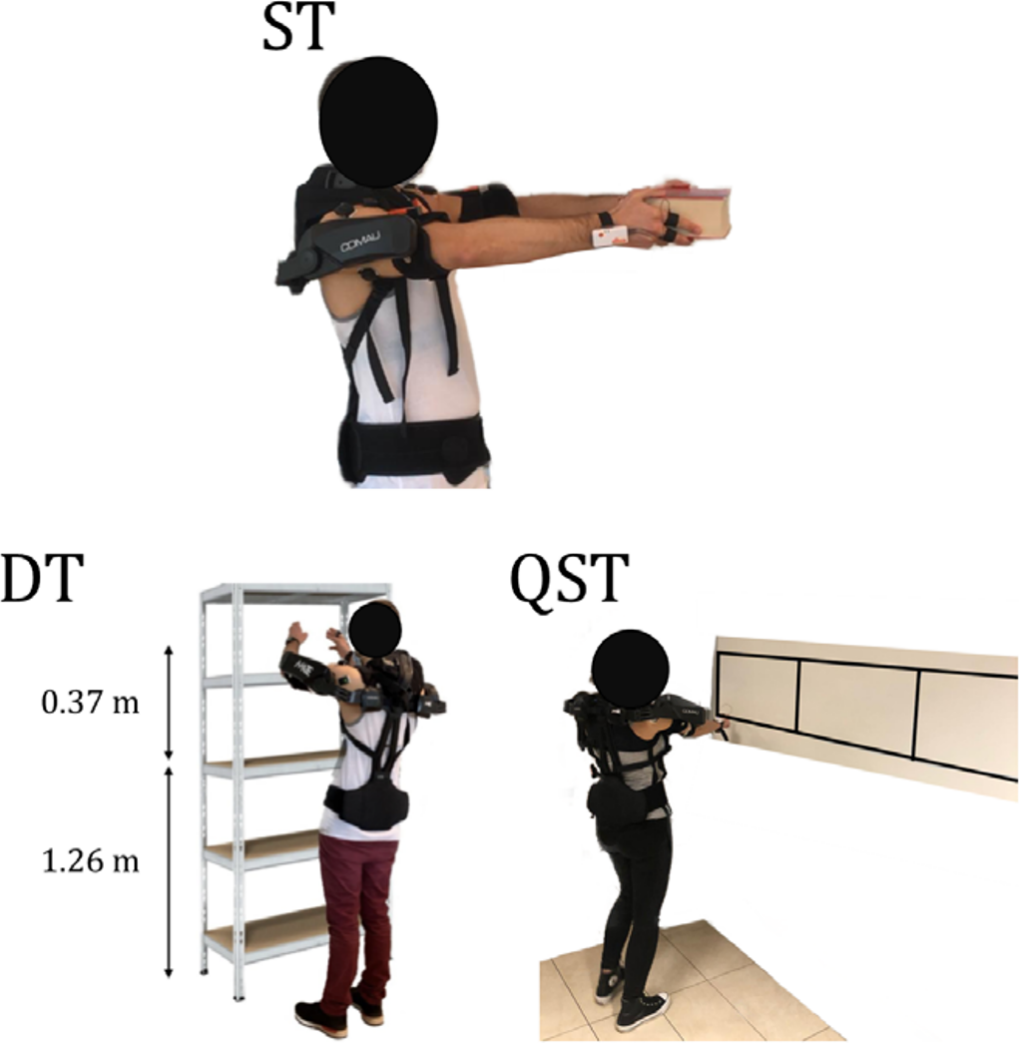
Exercises proposed in the experimental protocol.

At the beginning of each experimental trial, each volunteer underwent a 10-minute familiarization phase, during which they performed free body movements and experienced the assigned manufacturing tasks and a 2-hour rest phase between each tested condition. Moreover, a 4-minute baseline recording was conducted to assess the physiological state of the participants in their resting condition. This study was carried out in collaboration with Stellantis, and the tasks were carefully designed to replicate specific activities performed by workers on production lines, ensuring their industrial relevance. As illustrated in Figure 4, each participant was required to perform the following tasks in a randomized order, both without and with one of the passive exoskeletons (MATE or PAEXO):

- Static Task (ST): holding a load weighing 3 kg assuming a static posture, standing with the arms extended (90° with respect to the trunk), until the subject feels discomfort or fatigue. There is no minimum or maximum time limit. This task assesses endurance in a static posture, replicating scenarios where workers must hold vehicle parts in position for prolonged periods during assembly.
- Dynamic Task (DT): move the arms between two defined positions, placed in front of the subject at 1.26 and 1.63 m from the ground, maintaining a rate of 30 actions/minute for a maximum duration of 600 seconds. The test can be stopped before the deadline if the subject feels tired or uncomfortable. This task tests the ability of the user to maintain dynamic motion over time, reflecting tasks that require the continuous handling and movement of vehicle components.
- Quasi-static task (QST): reproduce a precision task with the subject standing with the shoulder and elbow flexed at 90°. The goal of the task is to reproduce the profile of a square wave with a duty cycle of 0.5 at 1 m from the ground for a maximum of 27 times. It reproduces a precision task, mirroring operations such as the application of sealants and adhesives, or quality inspection, which demand controlled movements and sustained positioning of the arms.

The experiments were randomized to minimize order effects, fatigue accumulation, and learning biases. This approach ensured a balanced distribution of potential fatigue influence across participants. Moreover, each participant was randomly assigned to test one of the exoskeletons. This approach was intended to reduce systematic biases for individual differences. Additionally, no participant had prior experience with either of the exoskeletons. This choice was to prevent the possibility of subjects learning the advantages and limitations of the first device, which could have influenced their performance and perception when testing the second exoskeleton. To ensure a comprehensive evaluation of exoskeleton performance, data were collected for both the dominant and non-dominant upper limbs. This choice was particularly relevant as the static task (ST) and dynamic task (DT) are bilateral tasks, requiring simultaneous use of both arms. This approach also helped determine whether the exoskeletons provided consistent support across both limbs or if their effects varied depending on limb dominance. Understanding these variations is essential for evaluating ergonomic benefits, movement coordination, and the potential risk of musculoskeletal imbalances during prolonged use. Therefore, muscle activation and kinematic data were recorded separately for each limb to capture task-specific asymmetries and the overall impact of the exoskeletons on bilateral movement dynamics.

#### 2.4.1. Participants and ethics

In this study, 16 volunteers have been involved, with no previous history of the trunk and/or upper limb orthopedic or neurological disorders. The experimental protocol was approved by the local Ethical Committee (Comitato Etico Fondazione Policlinico Campus Bio-Medico di Roma, reference number: 79/21 ComEt CBM). All subjects gave written informed consent to participate in the experiments, conducted in accordance with the Declaration of Helsinki. The participants were randomized into two groups and each of them has been asked to perform the tasks without (NO-EXO) and with one of the exoskeletons (PAEXO or MATE).

### 2.5. Performance indicators

This section outlines the performance indicators computed to assess the effect produced with the introduction of the passive upper limb exoskeleton.

From a kinematics point of view, the range of motion (RoM) of the *i*th joint angle is computed as(3)

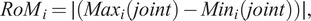

where 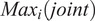 and 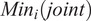 represent the maximum and the minimum *i*th joint angle exhibited in the *i*th complete repetition of the performed exercise. The standard deviation of the *i*th joint angle is computed during the trial to determine the angular deviation ( 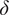 ) of each upper limb.

The time duration of the trial is noted in order to assess whether the exoskeleton use has an effect on the participants endurance.

Moreover, imaging EMG (iEMG) is computed in each condition to estimate the activation level of each muscle as follows:(4)

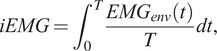

where 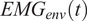 is the envelope of the EMG data and *T* is the duration of the task. The Dimitrov Index (DI) (Dimitrov et al., 2006) is computed to estimate the muscular fatigue level, as follows:(5)

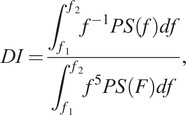

where 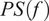 is the signal power spectrum and 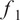 and 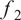 are the lowest and the highest frequency of the bandwidth. The DI is an indicator of muscle fatigue, evaluated for each muscle during its contraction phase, and increases as the level of muscle fatigue increases, as evidenced in the literature (Dimitrov et al., 2006; Gonzalez-Izal et al., 2012).

To assess the exoskeleton use impact on the physiological state of the participants, the physiological parameters extracted by means of the wearable sensing units are normalized with respect to the ones recorded in the baseline condition. In this way, it is possible to reduce the inter-subject and the sample size variability (Tamantini et al., 2021). Starting from the electrocardiographic (ECG) signal, the instantaneous HR and the root mean square of successive heartbeat differences, expressed in (ms), are computed as HRV metrics. The RR and the GSR of the users are also monitored.

The physiological parameters are normalized as follows:(6)

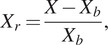

where 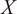 is one of each computed physiological parameter 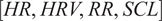 , 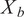 is the mean value of the physiological parameter collected during the baseline recording phase, and 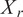 is the physiological response evoked in the participants during the execution of the proposed exercise. The raw galvanic skin response signal is post-processed to retrieve slowly changing components, the skin conductance level (SCL), by using a fourth-order Butterworth low-pass filter, with a cutoff frequency of 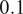 Hz (Novak et al., 2011; Cittadini et al., 2023).

To assess the user experience related to the use of passive upper limb exoskeleton use, two self-reported questionnaires were administered to the enrolled participants. They were the BORG scale (Borg, 1982) and the System Usability Scale (SUS) (Brooke, 1996). The first one, also called rate of perceived exertion (RPE), provides insight into the perceived physical strain during the execution of any physical activity. It ranges from 6 to 20, where 6 means no exertion at all and 20 means maximal exertion. While heart rate provides an objective physiological measurement of exertion, RPE offers a subjective assessment of perceived effort. This combination allows for a more comprehensive understanding of the physical demands during the tasks, as it highlights the relationship between perceived effort and physiological data. The RPE scale is commonly used in occupational settings and complements heart rate measurements, offering additional context to the assessment of the physical exertion. Moreover, the SUS index evaluates the perceived usability of the robotic device. Its score is calculated by adjusting the responses to its 10 items: for odd-numbered (positive) questions, 1 is subtracted from the score, while for even-numbered (negative) questions, the score is subtracted from 5. The adjusted scores are then summed and multiplied by 2.5, yielding a final score ranging from 0 to 100, where higher values indicate better usability.

### 2.6. Statistical analysis

To evaluate the effect of exoskeleton use, a statistical analysis has been carried out on the collected data. More in detail, an analysis of variance has been carried out using ANOVA, which makes it possible to compare more than two testing groups. The independent variables in this study were the use of the exoskeleton (No-EXO, EXO), the type of exoskeleton (MATE, Paexo), and the assigned task (ST, DT, QST). The Kruskal–Wallis test was also employed as a non-parametric alternative to assess the robustness of the results in case of deviations from normality. It is used to determine whether there are significant differences between the three investigated conditions: NO-EXO, PAEXO, and MATE. Since all the aforementioned comparisons are evaluated between three arrays, the significance level takes into account the Bonferroni correction for multiple comparisons at 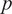 -value 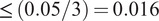 .

## 3. Results

A total of 16 volunteers (mean age: 14 M and 2 F, 26.9 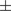 1.7 y.o., weight: 75.78 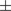 10.51 kg, height: 1.79 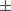 0.07 m) have been involved in this study. Table 1 presents the execution time for the Static Task (ST) for each participant. The average holding times recorded in the three conditions were 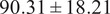 seconds, 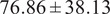 seconds, and 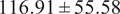 seconds for NO-EXO, PAEXO, and MATE, respectively.

**Table 1.** Execution time for the ST per subject

*N*	NO EXO (s)	PAEXO (s)	*N*	NO EXO (s)	MATE (s)
1	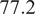	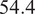	9	82.5	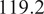
2	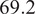	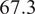	10	58.3	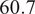
3	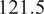	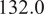	11	60.2	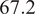
4	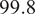	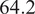	12	62.3	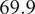
5	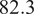	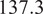	13	108.3	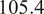
6	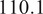	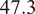	14	82.8	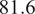
7	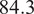	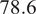	15	78.3	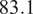
8	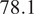	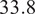	16	90.1	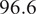
	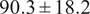	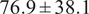		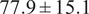	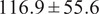

**Figure 5.**
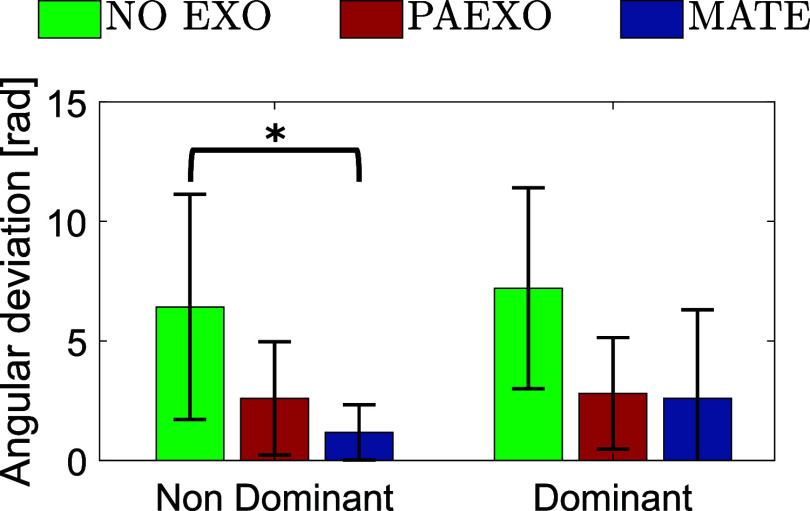
Mean angular deviation ( 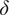 ) of the ST.

**Figure 6.**
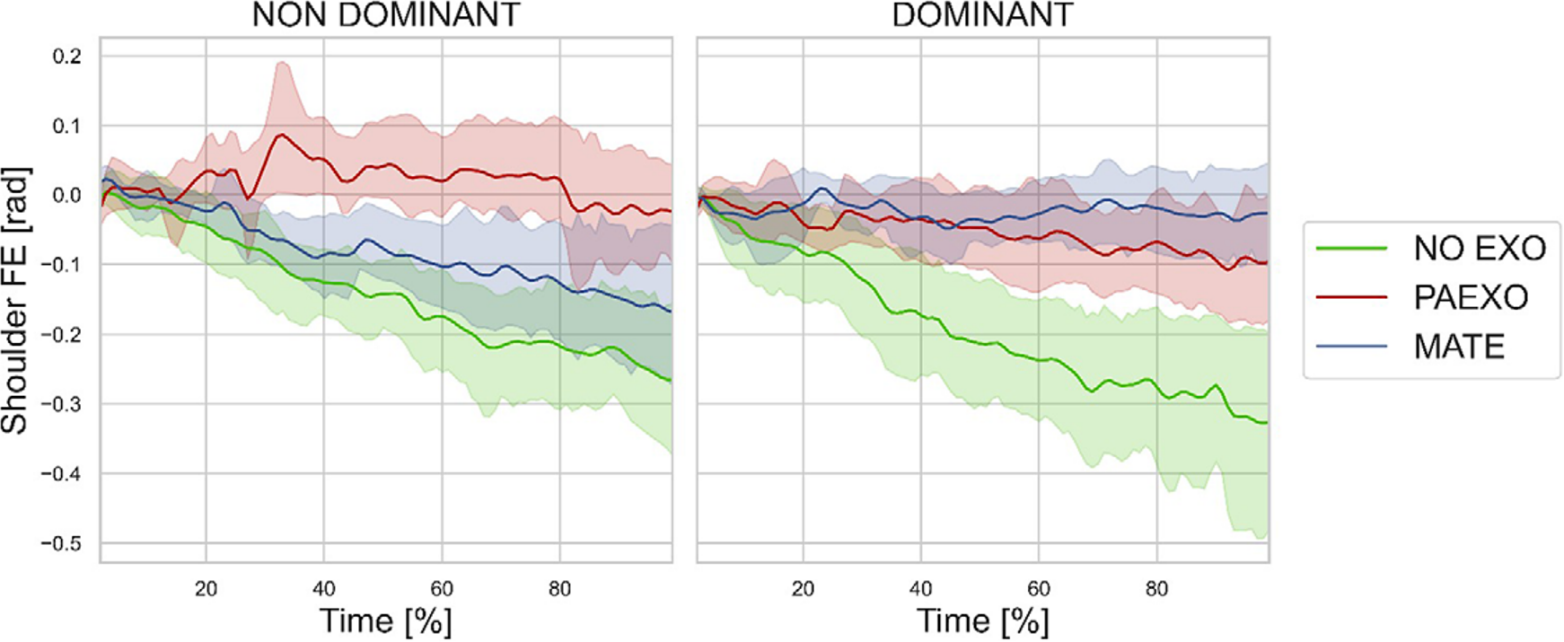
Mean and standard deviation of the shoulder flexion/extension angle estimated for ST in the three experimental conditions for dominant and non-dominant limb (green: NO-EXO, red: PAEXO, blue: MATE).

Figure 5 illustrates the average angular deviation 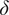 measured during the execution of the static task. A significant difference was observed in the non-dominant limb ( 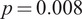 ), indicating a statistically relevant change in postural stability while using the exoskeletons. Figure 6 presents the shoulder flexion-extension (FE) angle over time for ST, comparing the NO-EXO, PAEXO, and MATE conditions for both the dominant and non-dominant limbs. The shaded regions represent the standard deviations across participants.

**Figure 7.**
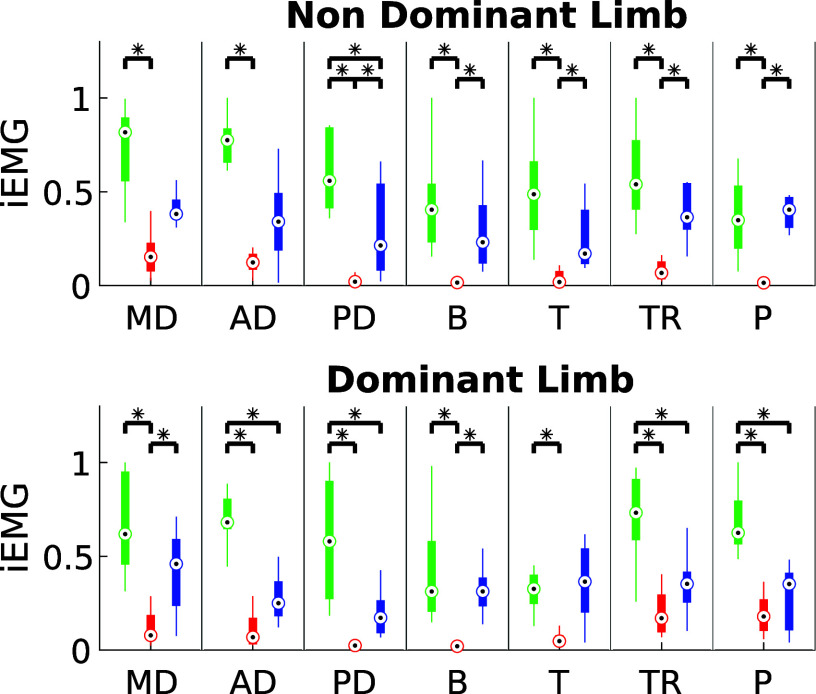
iEMG estimated for ST in the three experimental conditions for dominant and non-dominant limb (green: NO- EXO, red: PAEXO, blue: MATE).

**Figure 8.**
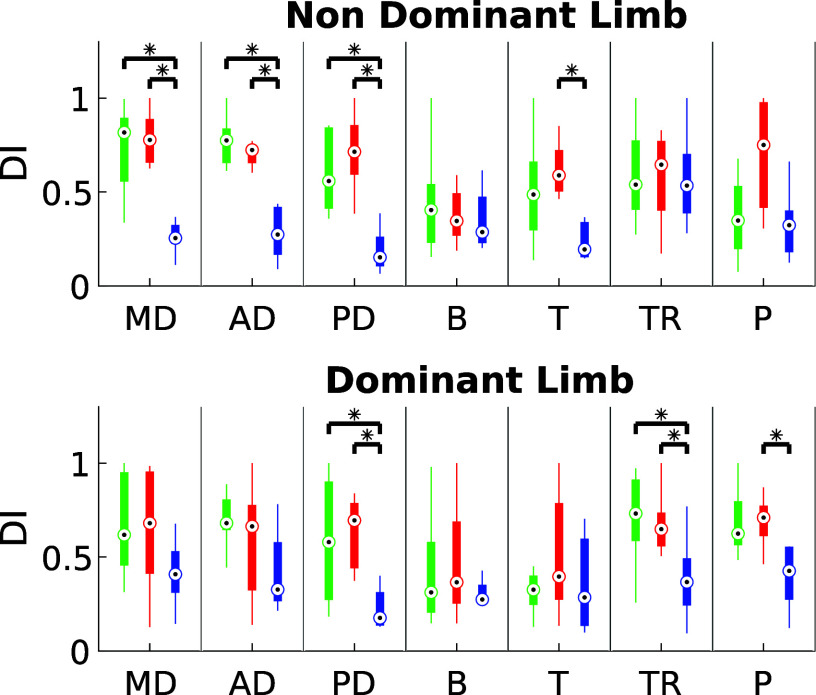
Dimitrov Index estimated for ST in the three experimental conditions for dominant and non-dominant limb (green: NO- EXO, red: PAEXO, blue: MATE).

The results about DI and iEMG for ST are shown in Figures 7 and 8, respectively. Both exoskeletons demonstrated a notable reduction in muscle fatigue, with improvements ranging between 20% and 60%, depending on the muscle group analyzed. Statistical analysis confirmed these findings ( 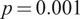 ), suggesting that the exoskeletons effectively alleviated muscle strain during static holding tasks.

**Figure 9.**
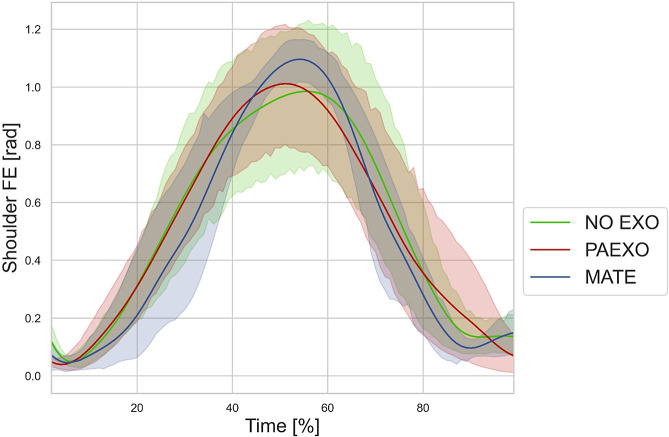
Mean and standard deviation of the shoulder flexion/extension angle estimated for DT in the three experimental conditions for dominant limb (green: NO-EXO, red: PAEXO, blue: MATE).

**Figure 10.**
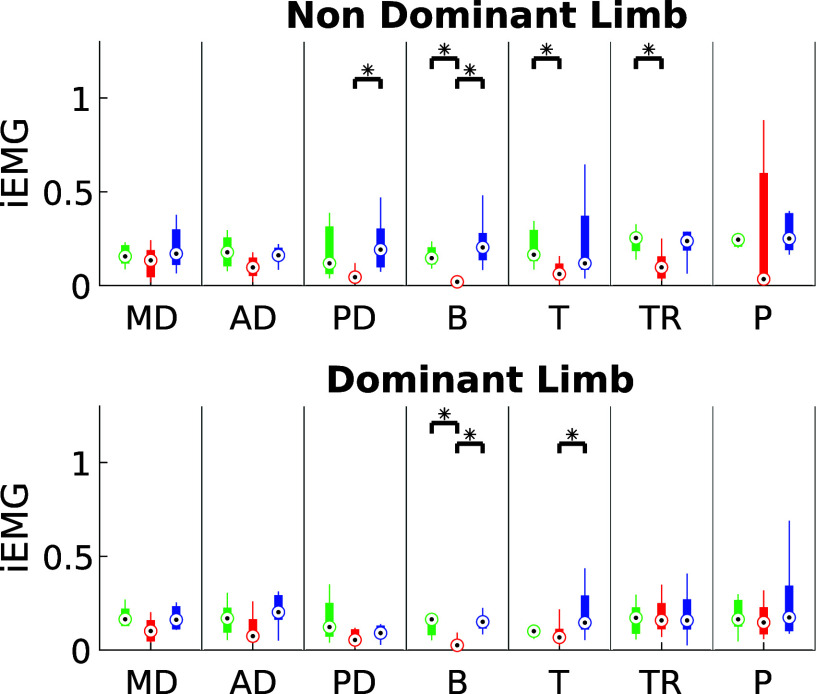
iEMG estimated for DT in the three experimental conditions for dominant and non-dominant limb (green: NO-EXO, red: PAEXO, blue: MATE).

Figure 9 illustrates the shoulder FE angle over time during the DT for the NO-EXO, PAEXO, and MATE conditions for the dominant limb. The data show the evolution of the movement cycle, highlighting differences in shoulder kinematics across the three conditions. Figure 10 reports the muscle activation levels recorded during the DT under the three experimental conditions, for both dominant and non-dominant limbs. The analysis highlighted differences in activation patterns between conditions, suggesting that exoskeletons may alter muscle recruitment strategies depending on the limb dominance.

**Figure 11.**
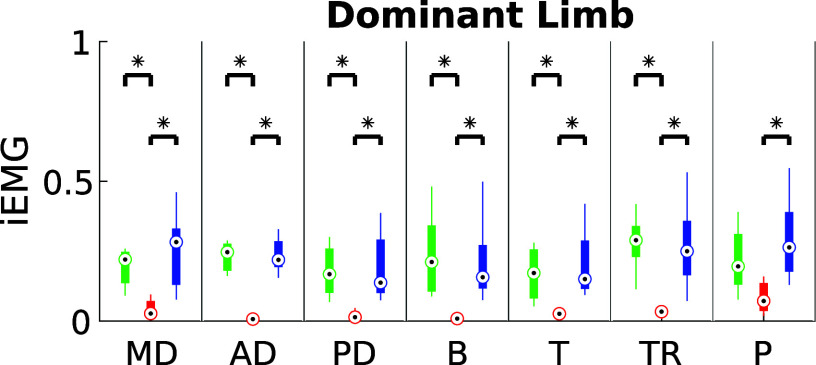
iEMG estimated for QST in the three experimental conditions for dominant limb (green: NO-EXO, red: PAEXO, blue: MATE).

Figure 11 presents the integrated EMG (iEMG) results for the Quasi-Static Task (QST) across the three experimental conditions. In this task, only the dominant limb was used and monitored. The results indicate that the use of exoskeletons influenced muscle activation patterns, although the specific effects varied depending on the device and task demands.

Moreover, Table 2 reports the mean values, along with the standard deviations of the RoM computed during the DT and QST exercises.

**Table 2.** Mean RoM of the DT and QDT

RoM (rad)	NO EXO	Paexo	MATE
DT	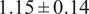	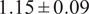	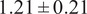
QST	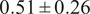	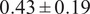	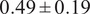

**Figure 12.**
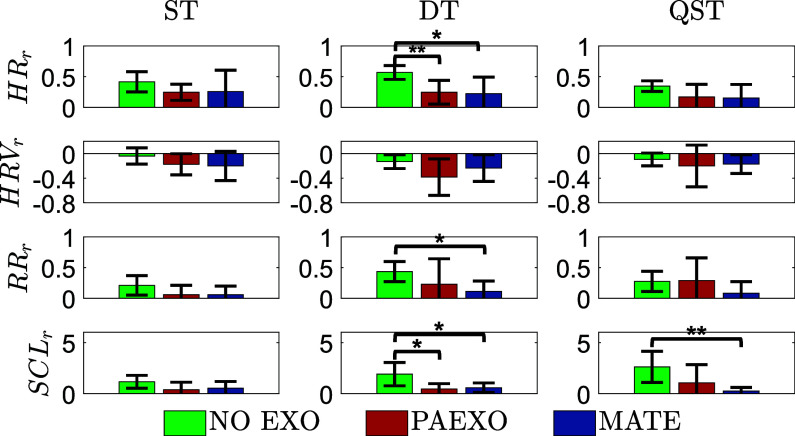
Physiological responses of the participants in all the experimental acquisitions.

The physiological responses of participants during all three tasks are displayed in Figure 12, in which bars represent mean values and the black line denotes standard deviation across participants. The data suggest measurable physiological differences between conditions, reflecting variations in workload and fatigue levels.

**Figure 13.**
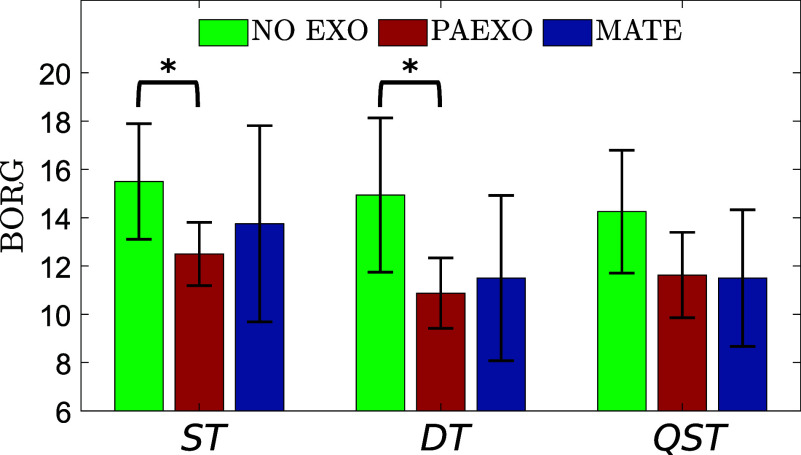
Borg scale scores collected for the three proposed exercises in the two experimental conditions.

The perceived exertion, rated by participants using the Borg scale, is summarized in Figure 13. The results indicate that exoskeleton use significantly influenced the perception of effort. Notably, the Paexo exoskeleton yielded significantly lower Borg scores compared to the NO-EXO condition in both ST ( 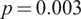 ) and DT ( 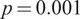 ), suggesting an improved user experience and reduced perceived strain. The SUS was administered to assess the subjective usability of the exoskeletons. The Paexo exoskeleton achieved a SUS score of 79.06 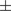 6.40, while the MATE exoskeleton obtained a lower usability score of 65.00 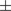 3.53. A statistically significant difference was found between the two devices ( 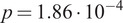 ), indicating that Paexo was perceived as more usable compared to MATE.

## 4. Discussions

The results of this study provide valuable insights into the biomechanical, physiological, and perceptual effects of using two passive upper limb exoskeletons, Paexo Shoulder and MATE, across different industrial tasks. The execution time for the ST was comparable between the NO-EXO and PAEXO conditions, while it increased when using the MATE exoskeleton. This suggests that the MATE exoskeleton provides greater support, allowing users to sustain load-carrying tasks for a longer duration. Additionally, inter-subject variability, particularly among sedentary workers, influenced performance, as differences in adaptation, task familiarity, and physical conditioning may have affected endurance levels. Moreover, both exoskeletons contributed to a reduction in angular deviation compared to the NO-EXO condition, with a statistically significant effect on the non-dominant limb ( 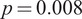 ), indicating that exoskeletons help users maintain a more stable posture, potentially reducing postural strain during prolonged static holding tasks. As shown in Figure 6, in the NO-EXO condition (green line), a progressive downward trend is observed in both limbs, reflecting a gradual loss of shoulder elevation over time due to muscular fatigue. In contrast, PAEXO (red line) and MATE (blue line) help maintain a more stable shoulder posture, particularly in the non-dominant limb, supporting the previous statistical findings. PAEXO allows for a higher shoulder position, particularly in the non-dominant limb, while MATE provides more consistent support across both limbs, including the dominant one. This suggests that MATE is particularly effective in this static task, as it better maintains shoulder positioning and reduces fatigue-induced postural decline, making it the more suitable option for prolonged static holding tasks. In the absence of an exoskeleton, muscle activity in the shoulder and elbow regions was significantly higher, as expected. Both exoskeletons contributed to a reduction in muscle activation, but the PAEXO exoskeleton demonstrated a greater ability to lower muscle activity across multiple muscle groups in both the dominant and non-dominant limbs, as confirmed by statistical analysis. An important observation emerges from the comparison between dominant and non-dominant limb activation during ST, reported in Figure 7. The MATE exoskeleton maintained normal coactivation of the non-dominant side, preventing a sharp decrease in muscle activation. This balance is crucial for joint stability, movement coordination, and the reduction of musculoskeletal imbalances or injuries, particularly in prolonged static postures. Moreover, the reduction in muscle activation (Figure 7) alongside increased fatigue (Figure 8) in the PAEXO condition can be explained by the nature of muscle fatigue and activation patterns. While PAEXO reduces overall muscle activation, it may alter the distribution of effort across muscle groups, leading to localized fatigue accumulation in specific muscles due to prolonged engagement in a static or semi-static posture. Additionally, muscle fatigue is not solely dependent on activation levels but also on sustained contractions, and biomechanical constraints introduced by the exoskeleton. Moreover, it is worth to note that both exoskeletons reduce fatigue in certain muscle groups, but PAEXO exhibits comparable fatigue levels to NO-EXO condition, also in the dominant limb. This suggests that, while PAEXO may reduce overall muscle activation, it could also lead to prolonged engagement of specific muscles, contributing to localized fatigue accumulation. Such findings highlight a significant reduction in muscle activity across nearly all muscle groups involved during static tasks, a finding that adds valuable insight to the existing literature. While previous studies have noted that exoskeletons can reduce shoulder muscle activity, the impact on muscle activation has often been task-dependent, with varying results for different muscle groups (De Bock et al., 2022; van der Have et al., 2022). For the Dynamic Task (DT), Figure 9 shows the shoulder flexion-extension (FE) trajectory during the Dynamic Task (DT) across the three conditions (NO-EXO, PAEXO, and MATE). Both exoskeletons generally follow the natural movement pattern, with MATE exhibiting a slightly higher peak flexion compared to PAEXO and NO-EXO. MATE provides greater support in the overhead phase, potentially reducing muscular effort during arm elevation. Conversely, PAEXO follows a trajectory closer to NO-EXO, indicating a lighter mechanical assistance. Furthermore, both exoskeletons effectively reduced muscle activation levels, yet an asymmetrical activation pattern was observed in the NO-EXO condition, creating an imbalance between the dominant and non-dominant sides. Interestingly, MATE appeared to better preserve the relationship between muscle activations, partially compensating for this imbalance. For the Quasi-Static Task (QST), which simulates a precision task, the MATE exoskeleton had a minimal effect on muscle activation levels. In contrast, PAEXO significantly reduced muscle activation compared to both NO-EXO and MATE conditions. This suggests that PAEXO may provide greater advantages in precision-based tasks requiring sustained arm positioning. It is worth mentioning that, to the best of current knowledge, there is a lack of extensive comparative analyses in the literature specifically exploring the effects of precision tasks alongside cognitive workload for manufacturing tasks, which leaves a gap in understanding the combined impact of these factors when using exoskeletons. Additionally, it is important to note that the exoskeletons did not hinder the participants’ range of motion, demonstrating that both devices allow users to complete tasks without excessive restrictions on movement. While the results of this study highlight the benefits of using passive exoskeletons in reducing muscle activation and fatigue, some potential drawbacks should also be considered. In particular, exoskeleton use altered muscle activation patterns, with Paexo demonstrating greater reductions in muscle activity across multiple muscle groups, while MATE better preserved coactivation on the non-dominant side. This balance is crucial for joint stability, movement coordination, and long-term musculoskeletal health, as excessive reductions in activation could lead to muscle deconditioning over prolonged use. Additionally, in dynamic tasks, muscle activation in the NO-EXO condition showed higher asymmetry between the dominant and non-dominant limbs, and while MATE partially mitigated this imbalance, some non-symmetrical activation patterns persisted. If not properly fitted or adjusted, exoskeletons could redistribute loads unevenly, potentially leading to compensatory movements, discomfort, or musculoskeletal strain. Furthermore, while both exoskeletons preserved the kinematic range of motion, the mechanical structure of MATE may restrict certain dynamic arm movements, making it less suitable for tasks requiring frequent repositioning of the arms.

All the experimental conditions induced physiological workload in the participants: as evident, the 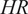 and 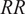 increased while 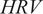 decreased during the task execution with respect to the physiological resting condition of the enrolled participants. The physiological responses collected in the ST are in line with all three experimental conditions. On the other hand, the other exercises highlighted relevant differences. During the DT the participants experimented very high physiological workload in the NO-EXO condition. Both exoskeletons are capable of reducing such influence. In particular, the impact on cardiac activity resulted to be significantly decreased when using the exoskeleton. The obtained 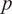 are 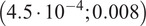 for the PAEXO and MATE condition, respectively. During the DT was also affected in a significant way the respiration, especially when the participants were wearing the MATE exoskeleton ( 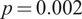 ). Physiological responses indicated that although exoskeletons reduced cardiovascular strain, MATE led to a significant increase in respiration rate, suggesting a potential increase in breathing effort. The physiological effects of passive exoskeletons are consistent with those observed in the literature. The use of exoskeletons led to an increase in heart rate, indicating additional exertion during activities. This phenomenon suggests a complex interaction where the device alleviates some physical strain while possibly introducing new metabolic challenges. The obtained findings align with this, as in the observed reduction in cardiovascular strain with exoskeleton use but also an increased respiratory effort in certain conditions. García et al. (2023) highlighted that physiological responses, such as heart rate, varied with task type and the presence of an exoskeleton. Similarly, those findings show a relationship between device usage and physical exertion, underscoring the importance of careful monitoring to ensure user safety and comfort during prolonged use. While exoskeletons, such as PAEXO and MATE, help mitigate musculoskeletal risks, they may elevate cardiovascular demands under certain conditions, especially when respiration is significantly impacted. For the cognitive workload, it emerges that the 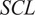 increased during the execution of the proposed exercises in the NO-EXO condition with respect to the other ones. It is worth observing that the autonomic responses were significantly reduced by the exoskeleton use during the DT ( 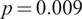 ). During the QST, in which the participants were asked to precisely follow the sealing pattern, the participants experimented a significantly higher cognitive workload in the NO-EXO condition with respect to the MATE use ( 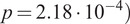 . The perceived effort, evaluated through the Borg scale, confirmed that Paexo significantly reduced perceived exertion compared to NO-EXO in both ST ( 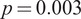 ) and DT ( 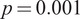 ), indicating improved comfort and reduced fatigue. Regarding usability, participants rated Paexo as significantly more usable than MATE, mainly due to its lighter weight. This is supported by the SUS results, where Paexo scored 79.06 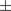 6.40, significantly higher than MATE (65.00 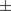 3.53, 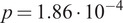 ). It is interesting to note that no similar findings related to cognitive workload have been reported in the existing literature on exoskeleton applications. These findings highlight the importance of device weight and ergonomics in user experience, particularly for prolonged use, suggesting that ergonomic design and comfort are critical factors in the long-term acceptance and usability of passive exoskeletons. However, MATE allowed for longer task durations in static load-holding tasks, as shown in Table 1, and resulted in lower fatigue levels, reported in Figure 8. From a general point of view, there are some key factors for the inter-subject variability, such as differences in muscle strength and endurance, affecting task performance and fatigue onset; adaptation to the exoskeleton, with some participants adjusting more quickly than others; and perceived discomfort and usability, where aspects such as comfort, fit, and perceived strain impacted the duration for which participants could maintain the position, even when muscle activation levels were reduced.

This study emphasizes the task-dependent nature of passive exoskeleton effectiveness. While both exoskeletons are designed to support repetitive upper limb tasks and tasks involving overhead arm positioning, their performance varies based on the specific task demands: MATE demonstrated better performance in static tasks, allowing users to sustain load-carrying positions for a longer duration with stable muscle coactivation. On the other hand, Paexo performed better in dynamic tasks, likely due to its lighter weight and greater freedom of movement, making it more suitable for tasks requiring frequent arm repositioning. Despite its effectiveness, MATE provided a more structured mechanical support, leading to a reduced level of muscle activation, which could be beneficial in tasks where fatigue management is critical. From a subjective perspective, both exoskeletons effectively reduced perceived muscle fatigue and were rated as ergonomically beneficial compared to the NO-EXO condition. It is also important to note that the study was conducted with sedentary workers, which may slightly influence the perceived exertion ratings. For such findings, a limitation of this study is that participants were divided into two groups, each assigned to a different exoskeleton, rather than using a within-subject design, in which all participants tested both devices. While this approach helped prevent learning effects and fatigue accumulation, it also introduced inter-individual variability, which may have influenced the results. To minimize this, we ensured that the two groups were demographically comparable and conducted within-group analyses to evaluate the effects of exoskeleton use relative to the NO-EXO condition. It is important to emphasize that the findings should be considered exploratory in nature, and further research using a within-subject design is useful to confirm such initial observations. Such findings highlight several critical design recommendations that can guide future iterations of passive wearable devices. The performance of both the PAEXO and MATE exoskeletons varied significantly depending on the task type. MATE demonstrated superior performance in static tasks by maintaining stable muscle coactivation and reducing fatigue during load-carrying tasks. However, its more structured mechanical support could potentially restrict some dynamic arm movements. In contrast, PAEXO provided better mobility and comfort for dynamic tasks due to its lighter weight, though it was less effective in maintaining postural stability during static tasks. This suggests that future exoskeleton designs should offer quantitative and customizable support levels, allowing adjustments based on the specific task requirements, such as heavier load-bearing tasks versus more dynamic, movement-intensive tasks. Moreover, exoskeleton designs should prioritize maintaining a balance between reducing muscle activation and preserving the natural activation patterns necessary for joint stability and movement coordination. This would help to ensure that users do not experience muscle deconditioning or other long-term negative effects associated with reduced muscle activation. Additionally, relying solely on objective metrics like muscle activation may fail to capture the user’s experience, especially concerning long-term comfort and fatigue. As demonstrated in this study, both exoskeletons reduced muscle activation, but subjective factors such as comfort and usability were crucial in determining their effectiveness in practical applications. Thus, a complete approach that integrates both objective and subjective data is essential for designing exoskeletons that meet the needs of users across various industrial tasks. Lastly, designers should consider the trade-off between reducing muscle activation and preserving natural movement patterns to avoid excessive reliance on certain muscle groups, which could lead to localized fatigue accumulation, as observed with PAEXO. Ergonomic comfort should also be prioritized, as PAEXO was rated more favorably for its lighter weight and comfort, which are key factors in ensuring the long-term acceptance and usability of exoskeletons in industrial environments.

## 5. Conclusions

In this article, the effects of two commercial upper limb passive exoskeletons have been assessed. More in detail, a monitoring system has been integrated to objectively assess the biomechanical and physiological effect of two commercial exoskeletons (that is, MATE and Paexo Shoulder) on the users during the execution of manufacturing tasks. The experimental sessions, carried out on 16 healthy volunteers, showed that the MATE allows a reduction in muscle load, ensuring the correct execution of the assigned task, especially for performing static tasks. The adoption of such systems has a significant effect in terms of muscle load, maintenance of the joint configuration, and reducing the effort perceived by the user. In DT, the best biomechanical performances were obtained with Paexo Shoulder. The obtained results push future works to a critical and comparative analysis of these systems on manufacturing workers to highlight any differences in muscular and psychophysiological effect during the execution of the task on the assembly line. Future developments will also be devoted to carry out tests in real scenarios on a population of workers with a higher average age to validate these findings and highlight any kinematic and muscular differences correlated to the adoption of exoskeletal devices. Moreover, future studies should consider adopting a crossover design, in which each participant tests multiple exoskeletons under controlled conditions, to reduce variability and strengthen the assessment of exoskeleton performance across different tasks.

## Data Availability

All data generated during and/or analysed in the current study are not openly accessible but are available from the corresponding author upon reasonable request.
